# Razor-type dermatomes enable quick and thin vaginal dissection with less bleeding in colpocleisis

**DOI:** 10.1007/s00192-019-04162-x

**Published:** 2019-11-27

**Authors:** Kumiko Kato, Yuji Hayashi, Mami Adachi, Ryota Ando, Hideji Kawanishi, Hirotaka Matsui, Takashi Kato, Hiroki Hirabayashi, Shoji Suzuki, Ryohei Hattori

**Affiliations:** 1 Department of Female Urology, Japanese Red Cross Nagoya First Hospital, 3-35 Michishita-cho, Nakamura-ku, Nagoya, 453-8511 Japan; 2 Department of Plastic Surgery, Japanese Red Cross Nagoya First Hospital, Nagoya, Japan; 3 Department of Pathology, Japanese Red Cross Nagoya First Hospital, Nagoya, Japan; 4 Department of Urology, Japanese Red Cross Nagoya First Hospital, Nagoya, Japan

**Keywords:** Pelvic organ prolapse, Total colpocleisis, Dermatome

## Abstract

**Introduction and hypothesis:**

Although colpocleisis is a low-invasive surgical option to treat pelvic organ prolapse, it sometimes involves a long operative time with substantial bleeding. To streamline the vaginal dissection process in colpoclesis, we introduced the usage of dermatomes.

**Methods:**

All patients were sexually inactive women with post-hysterectomy prolapse. Data of the dermatome group were retrospectively compared with those of the historical control group based on operative features, perioperative complications and pathology of dissected tissue. In the dermatome group, 34 women underwent total colpocleisis with vaginal dissection using dermatomes; 4 were done mainly with electric dermatomes, and 30 were done with razor-type dermatomes. In the control group, 20 women underwent total colpocleisis with vaginal dissection using Metzenbaum scissors.

**Results:**

Using dermatomes in vaginal dissection was helpful to shorten total operative time (including perineoplasty) by one third from 76 to 51 min, to shorten the time of colpocleisis by half, from 62 to 32 min, and to reduce intraoperative bleeding by 76%, from 62 to 15 ml. In addition, none in the dermatome group and 2/20 patients in the control group had unintended peritoneal opening. Dissection with scissors removed not only the epithelium and submucosal layer but also the muscle layer. This was minimized with razor-type dermatomes and never occurred with electric dermatomes. Whereas electric dermatomes are difficult to get accustomed to and are expensive, razor-type dermatomes enable thinner dissection compared with scissors, are easy to handle and are inexpensive.

**Conclusions:**

Razor-type dermatomes enable quick and thin vaginal dissection with less bleeding. Therefore, they can be recommended as a practical tool for colpocleisis, a prolapse operation mainly for frail elderly patients.

## Introduction

Colpocleisis has been regarded as an old-fashioned surgery for pelvic organ prolapse (POP), but it is gaining some popularity in rapidly aging societies as a good option for sexually inactive elderly patients. This obliterative procedure has consistently proven to be highly effective with an anatomical success rate > 90% [1–3]. Furthermore, it is reported to be associated with fewer adverse events than vaginal reconstructive surgery [4] and thus can be a safe, minimally invasive option for surgical treatment of POP.

However, despite the assumed low invasiveness, even colpocleisis sometimes involves a long operative time with more bleeding. This is because, to get proper tissue fusion, the vaginal epithelium must be thoroughly removed [5], and deeper dissection can damage larger blood vessels. In cases with post-hysterectomy prolapse, the vaginal wall tends to be thin; thus, vaginal dissection becomes difficult, and unintended opening of the peritoneum may occur.

To streamline the vaginal dissection process in colpocleisis, we introduced the usage of electric and razor-type dermatomes, which are the standard surgical instruments to produce thin slices of skin in plastic surgery for skin grafting and debridement to treat burns and injuries. Furthermore, we compared the operative time, intraoperative bleeding and thickness of dissection using these methods versus using scissors.

## Materials and methods

This study is a retrospective cohort study of sexually inactive women who underwent total colpocleisis for post-hysterectomy vaginal vault prolapse at a general hospital between 2015 and 2018. It was approved by the ethics committee of our institution. These operations were performed by a single urologist who had over 30 years of experience in female pelvic floor medicine.

At our institution, it is a long-standing standard practice to avoid concurrent anti-incontinence procedures even when patients have symptomatic stress urinary incontinence. On the other hand, concurrent perineoplasty was done based on the surgeon’s decision before 2015, and after that it became a standard practice.

**Fig. 1:**
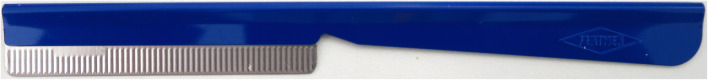
Razor-type dermatome (FEATHER Disposable Dermatome, FEATHER Safety Razor Co., Ltd.)

In the dermatome group, 34 women underwent total colpocleisis with vaginal dissection using dermatomes between March 2017 and May 2018: 4 done mainly with electric dermatomes (Zimmer Electric Dermatome, Zimmer Biomet Holdings, Inc.) and 30 done with razor-type dermatomes (FEATHER Disposable Dermatome, FEATHER Safety Razor Co., Ltd., Fig. 1). At first, we used electric dermatomes, which left many areas uncut, and it was necessary to use razor-type dermatomes to manage uncut tissue. After four cases, we decided to stop using electric dermatomes and to use only razor-type dermatomes for all the remaining cases. In the control group, 20 women underwent total colpocleisis with vaginal dissection using only Metzenbaum scissors during 2015 and 2017. There were no exclusion criteria, and all cases of total colpocleisis done in the described period of time were included in this study.

Following induction of spinal anesthesia and administration of preoperative antibiotics, patients were positioned in the dorsolithotomy position. A Foley catheter was inserted, and the position of the bladder neck was identified. Under hydrodissection with 1:1,000,000 diluted epinephrine saline, the vaginal wall was distended by pulling it with Allis forceps and pressing it forward from behind with gauzes. A circumferential superficial incision was made with a scalpel at the level of the bladder neck and 3 cm proximal to the perineum.

In the control group, a quadrant-based dissection [5] was performed using sharp dissection with Metzenbaum scissors. In the dermatome group, electric dermatomes, set at a depth of 0.5 mm and width of 2.5 cm, were handled by a trained plastic surgeon, and leftover epithelium was removed with razor-type dermatomes. Razor-type dermatomes were used by a urologist who had no experience with dermatomes. In both groups, the same method was used for serial purse-string sutures to obliterate the vaginal cavity, transverse closure of vaginal epithelium and perineoplasty. Operative time, intraoperative bleeding and perioperative complications were compared between the two groups. Pelvic examination and POP quantification (POP-Q) system analysis were done at baseline, 3 and 12 months after the operation. Anatomical recurrence was defined as the occurrence of POP-Q stage II or greater.

Pathology was investigated using H&E staining (hematoxylin and eosin staining) in vaginal specimens dissected with electric dermatomes, razor-type dermatomes and Metzenbaum scissors. The layer of dissection and thickness of dissection for the three methods were then compared.

All values were expressed using the mean ± standard deviation (SD) and analyzed with EZR, which is a graphical user interface for R [6]. The sample size calculation based on the significant change of operative time was conducted as follows. In a previous study of total colpocleisis [1], the standard deviation of operative time was approximately 30 min. We estimated the sample size with a change of mean operative time of 25 min (effect size of 0.8), power of 0.8, significance level of 0.05 and ratio of the control group to dermatome group of 1:1.5. A sample size of 19 in the control group and 29 in the dermatome group was needed. Data distribution was assessed with the Shapiro-Wilk test for continuous variables. As a result, age and BMI were normally distributed, and Student’s *t*-test was used for analysis. Since other continuous variables were not normally distributed, Mann-Whitney U test was used for analysis. For categorical variables, Fisher’s exact test was used to compare their proportions between groups. The significance level was set at *p* < 0.05.

## Results

Patient demographics and other baseline characteristics between the dermatome group (*n* = 34, mean age 76.0 years) and control group (*n* = 20, mean age 75.2 years) showed no significant differences (Table 1). All patients in the dermatome group and 18/20 patients in the control group underwent concomitant perineoplasty. Both operative time and bleeding significantly decreased in the dermatome group compared with the control group; mean total operative time including perineoplasty was 50.6 min vs. 76.4 min, operative time of colpoclesis was 31.9 min vs. 62.4 min, and intraoperative bleeding was 15.1 ml vs. 62.3 ml (Table 2). The ratio of patients who had bleeding ≥ 100 ml was significantly lower in the dermatome group than in the control group (Table 2). Hemostasis was basically not needed after dissection with dermatomes unlike with scissors during vaginal dissection. We found no significant differences in all data except the thickness of vaginal dissection between the electric dermatome subgroup and razor-type dermatome subgroup.

**Table 1:** Demographics of patients with post-hysterectomy incontinence

	Control group (*n* = 20)	Dermatome group (*n* = 34)	*p* value
Age, years, mean ± SD	75.2 ± 5.9	76.0 ± 6.4	0.649
Parity, mean ± SD	2.4 ± 0.5	2.4 ± 0.7	0.814
BMI, mean ± SD	24.2 ± 3.0	23.7 ± 3.3	0.567
Preoperative POP-Q stage, *n* (%)			0.262
III	6 (30.0)	16 (47.1)	
IV	14 (70.0)	18 (52.9)	
Method of previous hysterectomy, *n* (%)			0.262
Abdominal	12 (60.0)	26 (76.5)	
Vaginal	8 (40.0)	7 (20.6)	
Laparoscopic	0 (0)	1 (2.9)	
Reason for hysterectomy, *n* (%)			0.488
Uterine myoma	10 (50.0)	21 (61.8)	
POP	8 (40.0)	8 (23.5)	
Others	2 (10.0)	5 (14.7)	
Medical history, *n* (%)			
Asthma	0 (0)	2 (5.9)	0.525
Cardiovascular disease	2 (10.0)	3 (8.8)	1
Cerebrovascular disease	1 (5.0)	3 (8.8)	1
Diabetes mellitus	5 (25.0)	4 (11.8)	0.266

**Table 2:** Surgical data of total colpocleisis 10^−7^

	Control group (*n* = 20)	Dermatome group (*n* = 34)	*p* value
Concurrent procedures, *n* (%)			
Perineoplasty	18 (90%)	34 (100%)	0.133
Anti-incontinence procedures	0 (0%)	0 (0%)	1
Total operative time, min, mean ± SD	76.4 ± 17.2	50.6 ± 8.9	4.28 × 10^−7^
Colpocleisis time, min, mean ± SD	62.4 ± 21.3	31.9 ± 9.4	1.91 × 10^−7^
Blood loss, ml, mean ± SD	62.3 ± 51.3	15.1 ± 30.6	3.84 × 10^−6^
Bleeding ≥ 100 ml, *n* (%)	5 (25%)	1 (2.9%)	0.022
Thickness of vaginal dissection, mm, mean ± SD	6.18 ± 2.04	1.50 ± 0.71	7.42 × 10^−7^
Perioperative complications, *n* (%)			
Peritoneal opening	2 (10.0)	0 (0)	0.133
Blood transfusion	0 (0)	0 (0)	1
ICU admission	0 (0)	0 (0)	1
Return to operating room	0 (0)	1 (2.9)	1
Rehospitalization	2 (10.0)	0 (0)	0.133
Reoperation for recurrence, *n* (%)	2 (10.0)	2 (5.9)	0.622

**Fig. 2:**
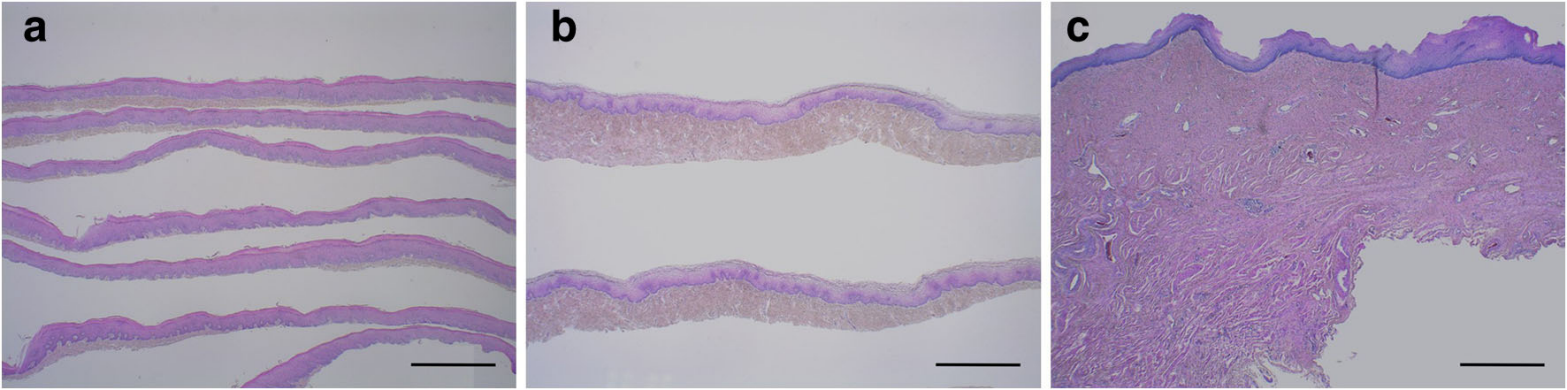
Pathological findings of vaginal specimens (H&E staining). Dissection with electric dermatomes (**a**), razor-type dermatomes (**b**) and Metzenbaum scissors (**c**). Scale bar = 1.0 mm

Pathological investigation revealed that there were statistically significant differences in the mean thickness of vaginal dissection with electric dermatomes 0.66 (± 0.29) mm, razor-type dermatomes 1.60 (± 0.67) mm and scissors 6.18 (± 2.04) mm (*p* < 0.01). Dissection with scissors resulted in the removal of not only the epithelium and submucosal layer but also the muscle layer. This was avoided or minimalized with razor-type dermatomes and never occurred with electric dermatomes (Fig. 2). Although electric dermatomes enabled the thinnest and most uniform dissection, due to the width of the blade, it was difficult to press the blade closely to the vaginal wall especially in cases with small prolapse.

No patients in the dermatome group and 2/20 patients in the control group had unintended peritoneal opening. One patient in the control group was rehospitalized and treated with antibiotics because of pyelonephritis and sepsis 17 days postoperatively. Another patient in the control group was rehospitalized 10 days postoperatively because of bleeding, which could be managed by vaginal gauze packing. In one case in the dermatome group, bleeding occurred at the edge of the incision on the same day, which was controlled by reoperation using only one stitch. No patients in either group had cases with blood transfusion, ICU admission, venous thromboembolism, urinary retention or death.

One year after the operation, 4/20 (20.0%) in the control group and 3/34(8.8%) in the dermatome group had anatomical recurrence. This study used a strict definition, regarding cases with POP-Q stage ≥ II as recurrence. Thus, patients with POP-Q stage II and no bothersome subjective symptoms were included in these recurrence rates. Of these, two (10%) in the control group and two (5.9%) in the dermatome group underwent reoperation for prolapse (*p* = 0.622). Regarding voiding dysfunction, 5/20 patients in the control group and 13/34 patients in the dermatome group had residual urine ≥ 100 ml preoperatively, which became < 60 ml after colpocleisis. One patient with complete urinary retention due to neurogenic bladder was managed with clean intermittent self-catheterization both before and after colpocleisis (catheterization techniques became easier after colpocloeisis). Regarding stress urinary incontinence (SUI), 5/20 patients in the control group and 6/34 patients in the dermatome group had mild SUI preoperatively. SUI remained mild or improved after colpocleisis, and neither group had cases necessitating second-stage anti-incontinence procedures.

## Discussion

To give a brief overview, this is the first report on a novel use of razor-type dermatomes to enable quick and thin vaginal dissection with less bleeding in colpoclesis. We introduced the usage of electric dermatomes following Dr. Takazawa’s presentation at the annual meeting of the Japanese Society of POP Surgery (JPOPS) in March 2017. However, due to technical difficulties, we promptly changed to razor-type dermatomes. When using electric dermatomes, it was difficult to press the blade close to the vaginal wall because of the width of the blade, and razor-type dermatomes were needed to manage uncut tissue. We analyzed the data of total colpocleisis in post-hysterectomy prolapse with or without the usage of dermatomes. Using dermatomes in vaginal dissection of colpocleisis was helpful to shorten mean total operative time (including perineoplasty) by one third from 76 min to 51 min and to shorten the time of colpocleisis by about half from 62 min to 32 min. In addition, it reduced intraoperative bleeding by 76%, from 62 ml to 15 ml. As the main targets of colpocleisis are frail elderly patients with multiple comorbidities, shorter operative time and less bleeding are beneficial for them. Although the mean intraoperative bleeding was not such a significant amount even in the control group, using dermatomes decreased it further and significantly decreased the ratio of patients with bleeding ≥ 100 ml. In addition, decreasing the operative time can be financially advantageous for the hospital and may shorten the waiting time for operations. We found no significant differences in these data between the electric dermatome subgroup and razor-type dermatome subgroup. Thus, the shift did not notably affect the results and conclusions of this study.

DeLancy et al. reported that mean operative time was 101 min and intraoperative bleeding was 206 ml in total colpocleisis, which was accompanied by concurrent anti-incontinence procedures in 17/33 and perineoplasty in 3/33 [1]. Crisp et al. wrote that mean operative time was 99 min and intraoperative bleeding was 100 ml, which contained both total colpocleisis (colpectomy) and partial colpocleisis. This was accompanied by concurrent operations such as perineoplasty (83.1%) and hysterectomy (10.8%) [7]. Hill et al. reported that mean operative time of colpectomy without concurrent hysterectomy was 108 min and intraoperative bleeding was 135 ml [8]. From these reports, we found an opportunity for improvement in the areas of operative time and intraoperative bleeding by introducing dermatomes in vaginal dissection.

To achieve proper tissue fusion in colpocleisis, it is desirable to remove all vaginal epithelium [5]. However, deeper dissection using usual methods with Metzenbaum scissors can damage larger blood vessels in the muscle layer, resulting in more bleeding. Mueller et al. reported that patients who underwent colpocleisis at high-volume centers had fewer complications and ICU admissions than those who underwent colpocleisis at low-volume centers [9], which suggests improved outcomes by individual surgeon volume [10]. Electric and razor-type dermatomes are originally intended for skin grafting and debridement in plastic surgery, and can efficiently dissect tissues thinly. Thus, using dermatomes can make the operation easier to perform for less experienced surgeons, shorten the dissection process and reduce the time for hemostasis.

Regarding the difference between the two types of dermatomes, although dissection was the thinnest and most uniform with electric dermatomes, they are difficult to become accustomed to and are expensive (machine $13,200, disposable blade $54). On the other hand, compared with scissors, razor-type dermatomes enabled thinner and more uniform dissection. Furthermore, they are easy to handle and inexpensive (disposable razor, $1.30). Therefore, when comparing these two types of dermatomes, razor-type dermatomes are the more practical choice.

Older women have more comorbidities than younger women, which leads to more perioperative complications. Sung et al. reported that elderly women aged > 80 years had 13.8 times increased mortality risk after surgical management of POP compared with women aged < 60 [4]. The risk of perioperative complications was also 1.4 times higher in elderly women aged > 80 years. Moreover, elderly women aged > 80 years who underwent obliterative procedures had a lower risk of complications than those who underwent reconstructive procedures (17.0% vs. 24.7%) [4]. In our study, about one third of the control and dermatome groups were aged > 80 years. Although neither group had cases with blood transfusion, ICU admission, venous thromboembolism, urinary retention or death, in the control group, we found perioperative complications in 4/20 (20.0%), two cases of unintended peritoneal opening and two cases of rehospitalization (one: pyelonephritis and sepsis, one: postoperative bleeding). However, in the dermatome group, we found perioperative complications in 1/34 (2.9%), i.e., one case of postoperative bleeding requiring reoperation on the same day. There were no cases of peritoneal opening or rehospitalizaion. In cases with post-hysterectomy prolapse, the vaginal wall tends to be thin and have scars and adhesions between the vaginal wall and peritoneum. Thus, thinner dissection using dermatomes was helpful to avoid peritoneal opening. Moreover, using dermatomes lowered invasiveness because of the shorter operative time and less bleeding without increasing recurrence. It may be argued that reoperation rates for recurrence (10% in the control group and 5.9% in the dermatome group) were high. However, this study was done in a referral center and included patients with poor conditions such as ischemic vaginal wall after multiple vaginal surgeries.

The disadvantage of colpocleisis is that it makes coital intercourse impossible. Therefore, despite its low invasiveness, surgeons are reluctant to offer obliterative procedures because of concern about patient regret. However, many papers have shown significant improvement of body image, QOL, bladder and bowel symptoms and a low level of regret and high satisfaction following colpoclesis [7, 11–13]. In our study, we included only elderly patients who had not had coital intercourse for at least several years and did not wish to have it in the future. We explained about other surgical options including colporraphy, transvaginal mesh repair and laparoscopic sarcocolpopexy, which preserve the ability to have coital intercourse. Having received this information, they still chose colpocleisis. For such patients, the loss of the ability to have coital intercourse did not have a negative impact.

The strengths of this study are that it is the first study to examine the use of dermatomes in the vaginal dissection process in colopocleisis and that it includes pathological examination. The limitations include the small number of patients and the fact that it is a retrospective study. Due to the apparent advantage of using dermatomes, we did not plan a prospective randomized controlled study. Although we also used dermatomes in partial colpocleisis (LeFort), we did not include these cases in this study in order to keep the inclusion criteria clear. Partial colpocleisis with dermatomes may become the subject of a future study. We are very cautious about doing partial colpocleisis in patients with uteruses because it can increase the risk of delaying the diagnosis of gynecological malignancies [14, 15].

In conclusion, razor-type dermatomes enable quick and thin vaginal dissection with less bleeding than scissors. They are easy to handle and cost-effective. Therefore, when performing colpocleisis, we recommend using razor-type dermatomes to minimize invasiveness and allow more precise manipulation.
